# Drug-induced loss of imprinting revealed using bioluminescent reporters of *Cdkn1c*

**DOI:** 10.1038/s41598-023-32747-6

**Published:** 2023-04-06

**Authors:** Andrew Dimond, Mathew Van de Pette, Victoria Taylor-Bateman, Karen Brown, Alessandro Sardini, Chad Whilding, Amelie Feytout, Rab K. Prinjha, Matthias Merkenschlager, Amanda G. Fisher

**Affiliations:** 1 Epigenetic Memory Group, MRC London Institute of Medical Sciences, Imperial College London, Hammersmith Hospital Campus, Du Cane Road, London, W12 0NN UK; 2 Whole Animal Physiology and Imaging, MRC London Institute of Medical Sciences, Imperial College London, Hammersmith Hospital Campus, Du Cane Road, London, W12 0NN UK; 3 Microscopy Facility, MRC London Institute of Medical Sciences, Imperial College London, Hammersmith Hospital Campus, Du Cane Road, London, W12 0NN UK; 4 Immunology and Epigenetics Research Unit, Research, GlaxoSmithKline, Gunnels Wood Road, Stevenage, SG1 2NY Herts UK; 5 Lymphocyte Development Group, MRC London Institute of Medical Sciences, Imperial College London, Hammersmith Hospital Campus, Du Cane Road, London, W12 0NN UK; 6 Present Address: MRC Toxicology Unit, University of Cambridge, Gleeson Building, Tennis Court Road, Cambridge, CB2 1QR UK

**Keywords:** Imprinting, Bioluminescence imaging, Drug screening

## Abstract

Genomic imprinting is an epigenetically mediated mechanism that regulates allelic expression of genes based upon parent-of-origin and provides a paradigm for studying epigenetic silencing and release. Here, bioluminescent reporters for the maternally-expressed imprinted gene *Cdkn1c* are used to examine the capacity of chromatin-modifying drugs to reverse paternal *Cdkn1c* silencing. Exposure of reporter mouse embryonic stem cells (mESCs) to 5-Azacytidine, HDAC inhibitors, BET inhibitors or GSK-J4 (KDM6A/B inhibitor) relieved repression of paternal *Cdkn1c*, either selectively or by inducing biallelic effects. Treatment of reporter fibroblasts with HDAC inhibitors or GSK-J4 resulted in similar paternal *Cdkn1c* activation, whereas BET inhibitor-induced loss of imprinting was specific to mESCs. Changes in allelic expression were generally not sustained in dividing cultures upon drug removal, indicating that the underlying epigenetic memory of silencing was maintained. In contrast, *Cdkn1c* de-repression by GSK-J4 was retained in both mESCs and fibroblasts following inhibitor removal, although this impact may be linked to cellular stress and DNA damage. Taken together, these data introduce bioluminescent reporter cells as tools for studying epigenetic silencing and disruption, and demonstrate that *Cdkn1c* imprinting requires distinct and cell-type specific chromatin features and modifying enzymes to enact and propagate a memory of silencing.

## Introduction

Epigenetic processes modulate the expression of genes without a change in DNA sequence. At least three distinct mechanisms contribute to epigenetic control, ensuring that gene ‘states’ are heritable: DNA methylation, non-coding (nc)RNAs and post-translational modifications of histones^1–7^. Other chromatin traits such as replication timing and the spatial proximity of genes to certain nuclear domains or structures, also correlate with gene expression although their importance for propagating epigenetic states remains uncertain^8–15^. In addition, in different organisms and at different genes, the contributions of DNA methylation, histone modifications and ncRNAs to gene regulation varies, showing functional redundancy. Genomic imprinting is an epigenetic phenomenon in which a gene shows differential expression based upon whether it was inherited from the mother or father^16–18^. Imprinted gene expression is therefore monoallelic in certain tissues and cell types and is reset in the germline. Differential expression of maternal and paternal alleles of imprinted genes provides an unrivalled opportunity to examine the mechanisms that underlie epigenetic gene silencing^16,17,19^, as well as challenges that lead to a loss of imprinting and allelic re-activation.

**Figure 1:**
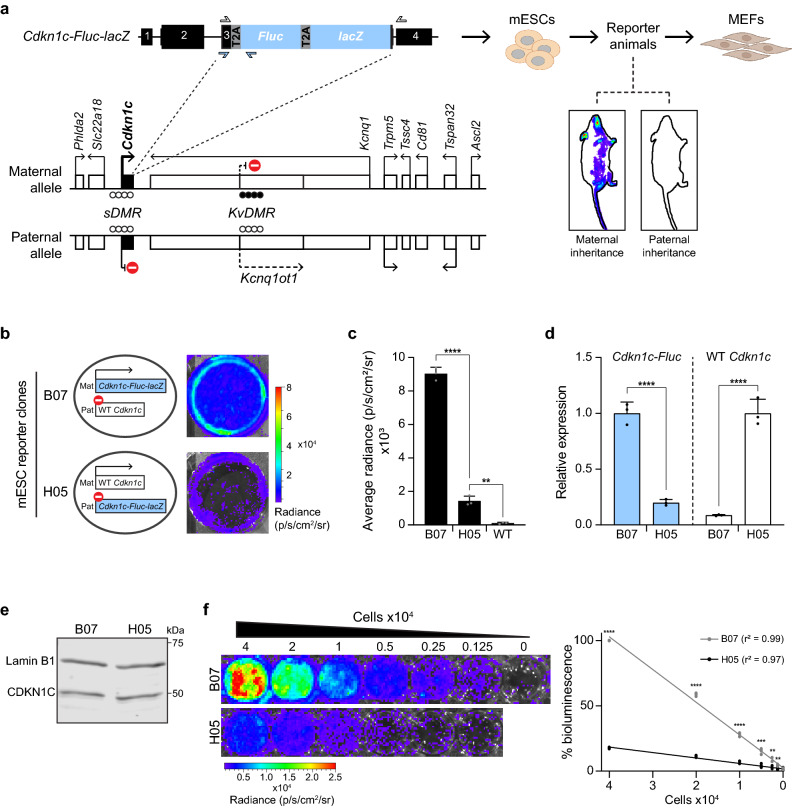
Bioluminescent imaging allows allelic readout of imprinted *Cdkn1c* expression. (**a**) Schematic illustrating the mouse *Cdkn1c* locus and bioluminescent reporter strategy (left), and its application in vitro and *in vivo*^55^ (right). Allelic gene expression and DMR methylation status at early development (closed circles = methylated CpGs, open circles = unmethylated CpGs) are shown for the locus. The *sDMR* is unmethylated on both alleles until E6.5^24^. The reporter construct, comprising *firefly luciferase* (*Fluc*), *β–galactosidase* (*lacZ*) and T2A sites, is inserted before the *Cdkn1c* stop codon in exon 3. Primers to distinguish expression of WT *Cdkn1c* and the reporter allele (*Cdkn1c-Fluc*) are shown by open and blue arrows respectively. Reporter mESCs were previously generated and used to create an in vivo model which exhibited proper imprinting of the reporter construct across generations (bioluminescent signal (blue) only present with maternal inheritance)^55^. Animals inheriting the reporter maternally or paternally can be used to derive corresponding reporter cells. Partially created with BioRender.com. (**b**) Schematic (left) and bioluminescent images (right, 6-well plate (wp)) of two mESC clones in which the reporter construct is inserted at either the maternal (clone B07) or paternal (clone H05) *Cdkn1c* allele. Since *Cdkn1c* is maternally expressed and paternally silenced, the reporter construct should be expressed in B07 cells and repressed in H05 cells, with the WT *Cdkn1c* allele exhibiting the reciprocal pattern. (**c**) Quantification of bioluminescent signal from B07, H05 and WT (no reporter) mESCs (n = 3; bars indicate mean; error bars represent standard deviation (SD); One-Way ANOVA p < 0.0001; results of Holm-Šídák’s multiple comparisons follow-up test are shown: ****p_adj_ < 0.0001, **p_adj_  = 0.0012). (**d**) Relative expression of *Cdkn1c-Fluc* (left) and WT *Cdkn1c* (right) alleles in B07 and H05 mESCs measured by RT-qPCR (n = 3; bars indicate geometric mean; error bars represent geometric SD; ****p < 0.0001, unpaired two-tailed t-tests on delta-Ct values). Primer positions to distinguish WT and reporter *Cdkn1c* alleles are illustrated in (**a**). (**e**) Western blot detection of CDKN1C protein in B07 and H05 mESCs using Lamin B1 as a loading control. The uncropped, unprocessed blot is available in the Supplementary Information file S1. (**f**) BLI of defined numbers of B07 and H05 mESCs in 48-well plates, showing representative images (left) and quantification (right). Quantification is relative to 4 × 10^4^ B07 mESCs for each dilution series (n = 4 independent dilution series; simple linear regression; multiple two-sided t-tests with Holm-Šídák’s correction comparing B07 to H05 for each dilution (****p_adj_ < 0.0001, ***p_adj_ < 0.001, **p_adj_ < 0.01).

*Cdkn1c* is a well-characterised maternally expressed, paternally silenced imprinted gene^20–27^ encoding a cyclin dependent kinase inhibitor which regulates foetal growth as well as placental and lineage-specific development^28–33^. Loss-of-function has been implicated in Beckwith-Wiedemann syndrome, whilst mutations and imprinting disruption have also been associated with IMAGe syndrome, Silver-Russell syndrome and cancer^34–38^. At the *Cdkn1c* locus (Fig. 1a), imprinting is primarily controlled by DNA methylation of the gametic differentially methylated region (*KvDMR*)^22,38–42^. In mice, a secondary somatic DMR (*sDMR*) overlapping the *Cdkn1c* promoter gains methylation during embryonic development (between E6.5 and E9.5) and is thought to stabilise and sustain paternal *Cdkn1c* silencing^24,43^. On the maternally-inherited *Cdkn1c* allele, *KvDMR* methylation prevents expression of a long ncRNA, *Kcnq1ot1*, allowing expression of *Cdkn1c*^39,44–46^. On the paternal allele, the *KvDMR* is unmethylated and *Kcnq1ot1* is expressed, leading to *Cdkn1c* silencing^44,45,47–49^. In addition to these features a variety of allelic differences, including histone modifications and CTCF binding, have been reported across the locus^23,49–54^, and these may also be important for gene regulation.

In order to study imprinting regulation and disruption, we previously generated bioluminescent mouse reporter lines for *Cdkn1c* and the paternally-expressed imprinted gene *Dlk1* by inserting the firefly luciferase gene (*Fluc*) into the endogenous loci^55,56^. Bioluminescent imaging (BLI) offers high sensitivity and extremely low background^57^, and provided allelic readouts of imprinted gene expression in vivo, throughout mouse lifespan and across generations. Using these tools, we showed that dietary challenges in utero could induce a prolonged loss of imprinting (LOI) in offspring. In contrast, injection of pregnant females with 5-Azacytidine and trichostatin A (TSA) provoked only transient *Cdkn1c* LOI, seen in embryos and perinatal offspring. This suggested that exposure to these drugs either partially (rather than permanently) relieved repression of the paternal allele, or that cells overexpressing *Cdkn1c* were selectively lost as animals matured. Epidrugs, such as TSA and 5-Azacytidine, represent a growing class of inhibitors, generated for eventual use in the clinic; however, challenges remain in selecting appropriate treatments, in understanding their cellular impacts, and in assessing the longevity of the responses which they elicit^58,59^.

In this study we examine *Cdkn1c*-reporter mouse embryonic stem cell (mESC) clones and mouse embryonic fibroblasts (MEFs) derived from these mice as complementary tools for in vitro investigation of epidrugs and epigenetic silencing. We show that BLI offers a convenient, sensitive and accurate readout of allelic gene expression in these cells, and allows the differential responses of maternal and paternal *Cdkn1c* to a panel of well-characterised chromatin-modifying drugs to be investigated. Using these tools, we identify several inhibitors which can induce LOI in one or both cell types, and examine whether this relief of epigenetic silencing is transient or sustained in cultured cells.

## Results

### Bioluminescent reporters for imprinted *Cdkn1c* expression

Previously, we generated a mouse reporter line for *Cdkn1c* in which the genes encoding Firefly luciferase (*Fluc*) and β-galactosidase (*lacZ*) were inserted into the 3’UTR of the *Cdkn1c* gene (Fig. 1a), using T2A sites to generate self-cleaving peptides from *Cdkn1c-Fluc-lacZ* mRNA^55^. In this earlier work we demonstrated that the *Cdkn1c-Fluc-lacZ* reporter showed the expected tissue-specific expression and was correctly imprinted in these mice (i.e. was maternally expressed) across successive generations, consistent with the insertion being non-disruptive and accurately reporting endogenous imprinted *Cdkn1c* expression^55^. In the current study, two heterozygous *Cdkn1c-Fluc-lacZ* mESC clones, that had been used to generate these animals, as well as MEFs derived from these mice (Fig. 1a), were examined in detail.

The two heterozygous *Cdkn1c-Fluc-lacZ* mESC clones originate from the same parental mESC line (TaconicArtemis C57BL/6N Tac), had comparable DNA methylation profiles (Fig. S1a), but differed with respect to reporter targeting into the maternal (B07) or paternal (H05) *Cdkn1c* allele (Fig. 1b). As anticipated, bioluminescence was readily detected in B07 cells upon luciferin addition (Fig. 1b, top) but was low in H05 cells (Fig. 1b, bottom), consistent with luciferase (and *Cdkn1c*) expression being largely restricted to the maternal allele. Although low, we could detect some bioluminescent signal in H05 cells, suggesting low-level expression from the paternal allele, consistent with previous mESC data^23^. The strong maternal bias and low-level paternal expression were confirmed by quantification of bioluminescence signal (Fig. 1c) and by reverse transcription quantitative real-time PCR (RT-qPCR) analysis (Fig. 1d) using primers which discriminate between targeted (*Cdkn1c-Fluc*) and wildtype (WT) *Cdkn1c* alleles (illustrated in Fig. 1a, white and blue arrows). These data confirmed that although overall expression of *Cdkn1c* and *Kcnq1ot1* transcripts (Fig. S1b) and CDKN1C protein (Fig. 1e) was indistinguishable between B07 and H05 clones, expression of *Cdkn1c-Fluc-lacZ* was much higher in B07 cells where insertion was on the maternal allele (Fig. 1d, left). In contrast, H05 cells expressed the maternally-derived WT allele (Fig. 1d, right), with paternal *Cdkn1c-Fluc-lacZ* largely repressed. The strong bias in expression between these genetically identical mESC clones provided us with an opportunity to investigate the chromatin elements that are required to maintain silencing of the paternal *Cdkn1c* allele and epidrugs which can disrupt normal imprinting.

To establish a robust cellular screening platform, we asked whether reporter-derived bioluminescence signal can provide a reliable estimate of allelic *Cdkn1c* expression levels by performing a dilution series of B07 and H05 cells. As shown in Fig. 1f, for both clones there is a highly linear relationship between bioluminescence signal and cell number (a proxy for expression level), with significant differences detectable between clones even at relatively small numbers of cells (as few as 1,250). These data indicate that bioluminescent measurements and comparisons can offer a robust and quantitative readout to screen for agents that induce the release of paternal silencing.

### Release of paternal *Cdkn1c* repression in mESCs treated with 5-Azacytidine or with HDAC, BET or KDM6A/B inhibitors

**Figure 2:**
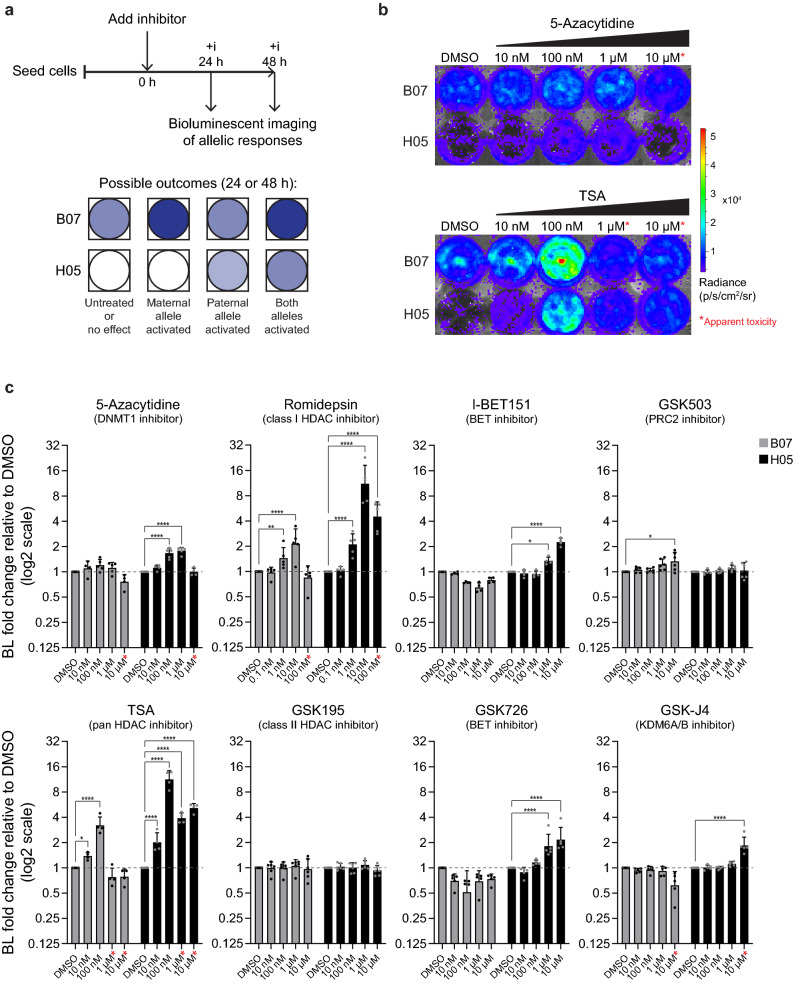
BLI identifies inhibitors causing loss of paternal *Cdkn1c* silencing in mESCs. (**a**) Experimental design to test the impact of inhibitors on allelic *Cdkn1c* expression measured by BLI (upper). Possible outcomes are illustrated below. B07 mESCs report maternal allele responses whilst H05 mESCs report paternal allele responses. (**b**) Bioluminescent images of B07 and H05 reporter mESCs treated for 24 h with different concentrations of 5-Azacytidine (5-Aza) or trichostatin A (TSA) (24-wp format). (**c**) Quantification of bioluminescent signal in B07 and H05 cells following 24 h treatment with the indicated inhibitors and concentrations, relative to DMSO vehicle control (24- or 48-wp format; n ≥ 3 independent experiments; bars indicate geometric mean; error bars represent geometric SD). Graphs are shown for a selected subset of the inhibitors tested, with quantification for the full panel provided in Table S1. A Two-Way ANOVA was performed using 24 h data (log2 transformed) for all inhibitors tested (Treatment (Inhibitor/mESC combination) p < 0.0001, Concentration p < 0.0001, Interaction p < 0.0001); significant increases relative to vehicle are indicated based on Holm-Šídák’s multiple comparisons follow-up test: ****p_adj_ < 0.0001, **p_adj_ < 0.01, *p_adj_ < 0.05. Red asterisks indicate treatments causing a visible reduction in cell number and/or apparent toxicity.

To examine the requirement for different chromatin modifications in maintaining silencing of paternal *Cdkn1c*, we treated H05 mESCs with drug inhibitors and examined luciferase activity relative to vehicle-treated controls after 24 and 48 h (Fig. 2a, upper panel). Studies performed in parallel using B07 cells enabled a comparison of drug responses at the maternal (active) allele. We reasoned that interventions might have different outcomes ranging from no effect on either allele, expression changes at both alleles (biallelic response), or selective increase in expression of the maternal allele (allelic activation) or the paternal allele (allelic de-repression). The predicted pattern of luciferase activity in clones B07 and H05 for these scenarios is illustrated in Fig. 2a (lower panel).

We screened a panel of 28 drugs (Supplementary Data S1) targeting a range of chromatin modifications including DNA methylation, histone acetylation and histone methylation, each over a 10,000-fold concentration range (Table S1). It is important to note that treatments which reduce cell growth or induce cell death will also result in reduced bioluminescent signal and may therefore lead to underestimates of reporter expression or activation. Although most drugs tested did not appear to significantly activate *Cdkn1c*-reporter expression in either mESC clone, a subset of inhibitors increased bioluminescence in one or both clones (Table S1), with drugs targeting the same pathways showing similar effects. For example, treatment with 5-Azacytidine, which inhibits DNMT1-mediated maintenance of DNA methylation at S-phase^60^, resulted in a small but statistically significant increase in bioluminescence in H05 cells, but not B07 cells (Fig. 2b, top panel; quantified in Fig. 2c, top left), suggesting that 5-Azacytidine selectively enhanced expression from the normally silent paternal allele. In contrast, treatment with TSA (a pan HDAC-inhibitor)^61,62^ provoked a marked increase in bioluminescence in both mESC reporter clones (Fig. 2b, lower panel; quantified in Fig. 2c, lower left), consistent with *Cdkn1c* upregulation from both maternal and paternal alleles, and in agreement with previous literature^63–65^. Interestingly, although similar responses were seen with the class I and IIa HDAC-inhibitor valproic acid (VPA)^66^ (Table S1) and the class I HDAC inhibitor Romidepsin^67^ (Fig. 2c, upper left middle), treatment with a class IIa HDAC inhibitor (GSK195)^62^ had no impact on *Cdkn1c* expression (Fig. 2c, lower middle left). We also observed selective, significant re-expression of paternal *Cdkn1c* in H05 cells following exposure to the BET inhibitors I-BET151^68–70^ or GSK726^71^ (Fig. 2c, middle right), and a similar trend was seen with a third BET inhibitor GSK0858 (Table S1). Inhibition of polycomb repressive complex 2 (PRC2)^72–74^ (which tri-methylates H3K27) caused a slight activation of maternal *Cdkn1c* (Fig. 2c, upper right), but none of the three PRC2 inhibitors tested induced bioluminescence increases in H05 cells (Fig. 2c, upper right and Table S1). In contrast, inhibition of KDM6A/B^75^ (enzymes which demethylate H3K27me3) with 10 µM GSK-J4 produced an increase in H05 bioluminescence (Fig. 2c, lower right), consistent with paternal *Cdkn1c* de-repression, although we observed visibly reduced cell numbers and apparent toxicity at this concentration making it harder to interpret the level of activation and allelic specificity. We also demonstrated that BLI has the potential to be scaled up to higher-throughput screening approaches. Using black-walled 96-well plates we could detect differences between B07 and H05 mESCs for as few as 500 cells (Fig. S2a). Furthermore, in pilot screens in this format, nine selected inhibitors yielded similar results to those seen in larger plate formats (Fig. S2b), including H05 responses to 5-Azacytidine, class I HDAC inhibitors, BET inhibitors and GSK-J4.

We verified our observations with 5-Azacytidine, TSA, I-BET151 and GSK-J4, which all appeared to de-repress paternal *Cdkn1c*, in follow-up BLI experiments (Fig. S3a) and measured the impact on viable cell numbers (Fig. S3b). TSA and GSK-J4 treatments caused a significant reduction in viable cells (either due to toxicity or reduced cell growth), raising the possibility that BLI may underestimate reporter activation for these treatments. We further validated our findings at the transcript level by RT-qPCR (Fig. S3c). RT-qPCR measurements confirmed that treatment with 5-Azacytidine or I-BET151 increased low level paternal expression, although this was not sufficient to significantly raise overall levels of *Cdkn1c* transcripts. In contrast, TSA and GSK-J4 treatments both increased total *Cdkn1c* expression, with contributions from maternal and paternal alleles. These data also confirmed that bioluminescence signal had underestimated reporter activation by GSK-J4, as a consequence of the drug’s impact on cell viability. Nonetheless, BLI screening had successfully identified this inhibitor as an activator of paternal *Cdkn1c*. Importantly, these RT-qPCR experiments also demonstrated that the WT *Cdkn1c* allele behaves similarly to the *Cdkn1c-Fluc-lacZ* allele, excluding the possibility that our observations are unique to the reporter construct.

To determine whether these chromatin-modifying drugs affect *Cdkn1c* expression by regulating *Kcnq1ot1* ncRNA (associated with paternal *Cdkn1c* silencing), we assessed expression of this transcript in treated B07 and H05 cells (Fig. S3d, left). *Kcnq1ot1* expression was not significantly altered by 5-Azacytidine, TSA, or BET inhibitor treatment, although GSK-J4 appeared to provoke a small activation. Exposure of mESCs to each of these drugs did however result in decreased expression of *Oct4* (Fig. S3d, right), consistent with compromised pluripotency. However, the extent of *Oct4* decrease does not appear to correspond to allelic *Cdkn1c* behaviour, indicating that *Cdkn1c* de-repression or activation is not solely explained by cellular differentiation.

### Temporary and sustained reactivation of paternal *Cdkn1c* in mESCs

**Figure 3:**
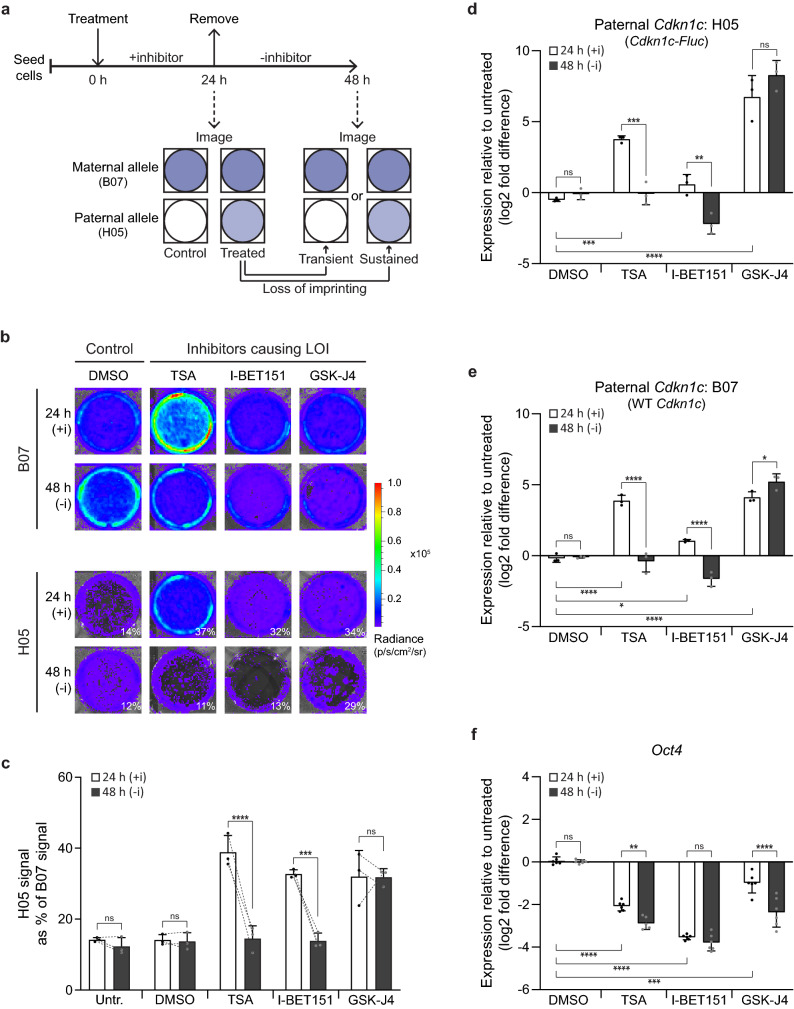
Inhibitors can induce temporary or sustained *Cdkn1c* LOI in mESCs. (**a**) Experimental design to test whether inhibitors induce temporary or sustained LOI (upper), with possible allelic responses illustrated below. Maternal *Cdkn1c* responses are reported by B07 mESCs whilst paternal *Cdkn1c* responses are reported by H05 mESCs. (**b**) B07 (upper) and H05 (lower) reporter mESCs imaged after 24 h treatment with inhibitors causing *Cdkn1c* LOI (100 nM TSA, 10 µM I-BET151, 10 µM GSK-J4), and re-imaged following inhibitor removal (48 h) (6-wp format). Images are representative of three independent experiments; percentages indicate H05 signal (representing paternal *Cdkn1c*) as a percentage of B07 signal (representing maternal *Cdkn1c*). (**c**) BLI quantification of relative paternal *Cdkn1c* activation in control conditions or following inhibitor treatment (24 h) and removal (48 h). Relative paternal allele contribution is shown by plotting H05 signal (representing paternal *Cdkn1c*) as a percentage of B07 signal (representing maternal *Cdkn1c*) (6-wp; n = 3 independent replicates; bars indicate mean; error bars represent SD; dotted lines link repeated measurements at 24 h and 48 h within replicates; Repeated Measures Two-Way ANOVA (Inhibitor x Time p = 0.0001, Inhibitor p < 0.0001, Time p < 0.0001, Well replicate p = 0.4593); results are shown for Holm-Šídák’s multiple comparisons follow-up test comparing 24 h with 48 h: ****p_adj_ < 0.0001, ***p_adj_ < 0.001, ns = not significant). (**d**,**e**) Confirmation of BLI results using RT-qPCR to measure paternal *Cdkn1c* expression in H05 mESCs ((**d**), *Cdkn1c-Fluc-lacZ*) or B07 mESCs ((**e**), WT *Cdkn1c*) following inhibitor addition (24 h), or addition and removal (48 h). Expression is plotted as log2 fold difference relative to untreated cells, normalised to *β-Actin* (n = 3 independent replicates; bars indicate mean; error bars represent SD; Two-Way ANOVAs ((**d**) H05 (Inhibitor p < 0.0001, Time p = 0.0025, Interaction p < 0.0001), (**e**) B07 (Inhibitor p < 0.0001, Time p < 0.0001, Interaction p < 0.0001); Holm-Šídák’s multiple comparisons follow-up tests comparing all means: significant 24 h changes vs DMSO and results of 24 h vs 48 h comparisons are shown: ****p_adj_ < 0.0001, ***p_adj_ < 0.001, **p_adj_ < 0.01, *p_adj_ < 0.05, ns = not significant). (**f**) RT-qPCR analysis of *Oct4* expression in reporter mESCs following inhibitor addition (24 h), or addition and removal (48 h). Expression is plotted as log2 fold difference relative to untreated cells, normalised to *β-Actin* (n = 6 (3 B07 and 3 H05 independent replicates combined); bars indicate mean; error bars represent SD; Two-Way ANOVA (Inhibitor p < 0.0001, Time p < 0.0001, Interaction p = 0.0002); Holm-Šídák’s multiple comparisons follow-up test comparing all means: significant 24 h changes vs DMSO and results of 24 h vs 48 h comparisons are shown: ****p_adj_ < 0.0001, ****p_adj_ < 0.001, **p_adj_ < 0.01, ns = not significant).

To determine whether the reactivation of paternal *Cdkn1c* is heritable or transient in dividing mESC cultures, we established a regime (illustrated in Fig. 3a) to treat B07 and H05 mESCs with chromatin-modifying drugs for 24 h (to induce LOI), before removing the drugs and re-imaging cells at 48 h. Comparing the ratio of bioluminescent signal in H05 versus B07 mESCs provides an estimate of the relative impact of each treatment on *Cdkn1c*-reporter expression from the paternal or maternal allele (Fig. S4a), whilst simultaneously accounting for impacts on cell number. By this measure, TSA, I-BET151 and GSK-J4 significantly shifted the ratio towards H05 cells, indicative of relative paternal activation (Fig. S4a), and this formed the basis for selecting these treatments to study the stability of their effects. In untreated or DMSO-treated mESCs, the ratio was unchanged between 24 and 48 h (drug treatment and removal; Fig. 3b,c). Exposure to TSA or I-BET151 resulted in paternal *Cdkn1c* upregulation and apparent loss of *Cdkn1c* imprinting (24 h), but this was significantly reversed after drug removal (48 h) (Fig. 3b,c). In contrast, the shift in H05/B07 ratio caused by GSK-J4 treatment was sustained in the absence of the drug (despite an overall reduction in signal due to apparent toxicity).

RT-qPCR analysis of *Cdkn1c* expression in B07 and H05 cells at 24 and 48 h (drug treatment and removal; Figs. 3d,e and S4b) confirmed the results obtained by BLI and verified that GSK-J4 had an impact on *Cdkn1c* expression and imprinting that, uniquely, appeared to be maintained after drug removal. The previously noted small increase in *Kcnq1ot1* expression triggered by GSK-J4 was also maintained at 48 h following drug removal (Fig. S4c). However, a paucity of cells in GSK J4-treated cultures was noted at 48 h, which raised the possibility that KDM6A/B inhibition does not support mESC division or is toxic. In all cases *Oct4* expression remained decreased (or decreased further) following inhibitor removal (Fig. 3f), suggesting that temporary or sustained release of paternal *Cdkn1c* silencing is not directly coupled to differentiation state.

### Fibroblasts exposed to TSA or inhibitors of KDM6A/B express paternal *Cdkn1c*

**Figure 4:**
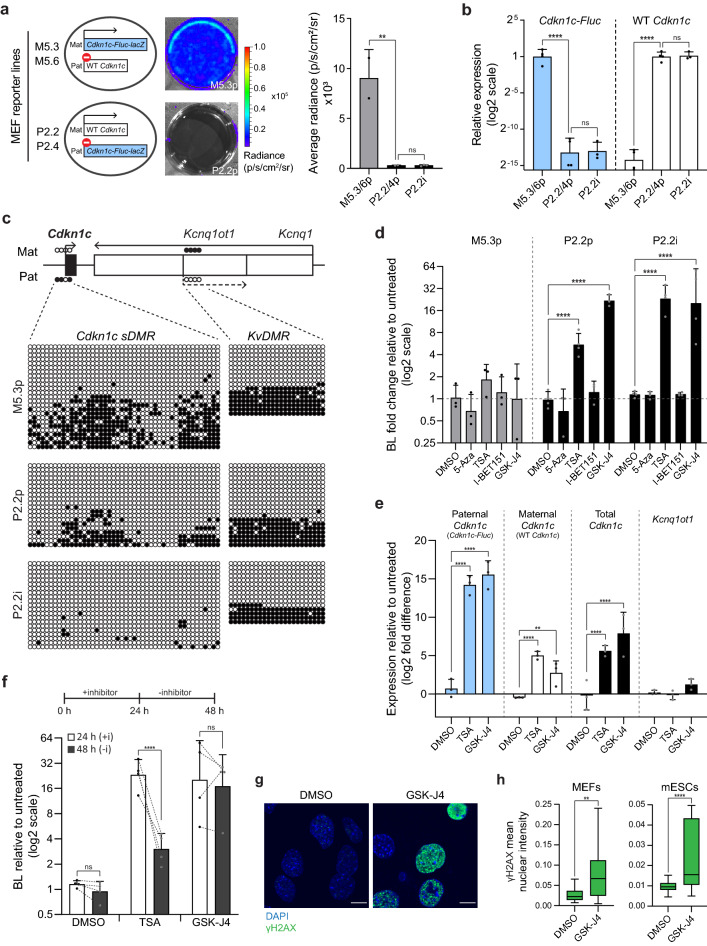
BLI of new MEF reporter lines highlights cell-type similarities and differences in *Cdkn1c* imprinted silencing. (**a**) Schematic (left) of MEF reporter lines derived from E13.5–14.5 embryos inheriting *Cdkn1c-Fluc-lacZ* maternally (M5.3 and M5.6, reporter expressed) or paternally (P2.2 and P2.4, reporter silenced). Primary MEF lines (‘p’) and immortalised P2.2 cells (P2.2i) were analysed by BLI. Representative primary MEF images (middle) and BLI quantification (right) are shown (6-wp; n = 2 (primary) or 3 (immortalised); bars indicate mean; error bars represent SD; One-Way ANOVA p = 0.0049; results of Holm-Šídák’s multiple comparisons follow-up test are shown for preselected pairs: **p_adj_ = 0.0073, ns = not significant). (**b**) Relative expression of *Cdkn1c-Fluc-lacZ* (left) and WT *Cdkn1c* (right) alleles in primary and immortalised MEF reporter lines measured by RT-qPCR (n = 4 (primary) or 3 (immortalised); bars indicate geometric mean; error bars represent geometric SD; One-Way ANOVAs on delta-Ct values (both p < 0.0001); results of Holm-Šídák’s multiple comparisons follow-up tests are shown for preselected pairs: ****p_adj_ < 0.0001, ns = not significant). (**c**) Expected *Cdkn1c sDMR* and *KvDMR* methylation patterns at E13.5–14.5^24^ (top) and bisulphite methylation analysis in derived reporter MEFs (below). Each row represents an individual clone (closed circles = methylated CpGs, open circles = unmethylated CpGs). Data for P2.2i are representative of two independent experiments. All reporter MEFs show the expected *KvDMR* methylation pattern (strands unmethylated or fully methylated). Primary MEFs (P5.3p and P2.2p) show partial *sDMR* methylation as expected; however, immortalised MEFs (P2.2i) lose *sDMR* methylation. (**d**) Quantification of bioluminescent signal in MEF reporter cells following 24 h treatment with the indicated inhibitors, relative to untreated cells (48-wp; n ≥ 3 independent replicates; bars indicate geometric mean; error bars represent geometric SD). Data is shown for 10 µM treatments, quantification for all concentrations tested is provided in Table S2. A Two-Way ANOVA was performed using data (log2 transformed) for all conditions tested (Treatment p < 0.0001, MEF line p < 0.0001, Interaction p < 0.0001); results of Holm-Šídák’s multiple comparisons follow-up test comparing to DMSO are shown: ****p_adj_ < 0.0001). (**e**) RT-qPCR analysis of P2.2i MEFs following 24 h treatment with the specified inhibitors (10 µM). Expression of the indicated targets is plotted as log2 fold difference relative to untreated cells, normalising to *β-Actin* (n = 3 independent replicates; bars indicate mean; error bars represent SD; Two-Way ANOVA (Treatment p < 0.0001, Gene p < 0.0001, Interaction p < 0.0001); significant results of Holm-Šídák’s multiple comparisons follow-up test comparing to DMSO are shown: ****p_adj_ < 0.0001, **p_adj_ < 0.01). (**f**) Paternal *Cdkn1c* activity in P2.2i MEFs was monitored by BLI following inhibitor treatment and removal (illustrated above). Bioluminescence signal was quantified relative to untreated cells at each timepoint (n = 4 independent replicates; bars indicate geometric mean; error bars represent geometric SD; dotted lines link repeated measurements at 24 h and 48 h within replicates; Repeated Measures Two-Way ANOVA on log2 transformed values (Inhibitor × Time p = 0.001, Inhibitor p = 0.0001, Time p = 0.0005, Well replicate p = 0.0157); results are shown for Holm-Šídák’s multiple comparisons follow-up test comparing 24 h with 48 h: ****p_adj_ < 0.0001, ns = not significant). (**g**) Representative images of γH2AX staining (green) in P2.2i MEFs treated for 24 h with DMSO or 10 µM GSK-J4. Images are representative of two independent experiments. Scale bars represent 10 µm. (**h**) Quantification of γH2AX nuclear staining intensity in P2.2i MEFs (left) and H05 mESCs (right) treated for 24 h with DMSO or 10 µM GSK-J4 (n ≥ 30 cells per condition; boxplots show the median, interquartile range (IQR) and ± 1.5 × IQR (Tukey method); results of Mann–Whitney tests are shown: **p = 0.0023, ****p < 0.0001).

To investigate whether paternal *Cdkn1c* re-expression could be equivalently induced in somatic cells, in addition to pluripotent mESCs, we derived primary (‘p’) MEFs (Fig. 1a) in which *Cdkn1c-Fluc-lacZ* was inherited either maternally (M5.3 and M5.6 cells) or paternally (P2.2 and P2.4 cells) (Fig. 4a, illustrated left). As predicted, luciferase activity was detected in M5.3/M5.6p MEFs, but not in P2.2/P2.4p MEFs, nor in an immortalised (‘i’) line, P2.2i (Fig. 4a, images and quantified right). Differential expression of WT *Cdkn1c* and *Cdkn1c-Fluc-lacZ* alleles between these genetically identical MEF lines was verified by RT-qPCR (Fig. 4b), whilst all lines expressed similar levels of total *Cdkn1c* and *Kcnq1ot1* (Fig. S5a).

Bisulphite analysis confirmed the expected bimodal pattern of DNA methylation at the *KvDMR,* and in contrast to mESCs revealed partial methylation at the *sDMR* in primary MEFs (Fig. 4c), which is gained during early development^24^. However, *sDMR* methylation was largely absent following immortalisation (compare P2.2i with P2.2p), consistent with hypomethylation as a consequence of long-term culture^76,77^. Interestingly, this was not accompanied by activation of paternal *Cdkn1c* (Fig. 4a,b), indicating that, at least in vitro, *sDMR* methylation is not essential for maintaining imprinted silencing in differentiated cells.

To compare *Cdkn1c* responses in pluripotent and differentiated cells we treated MEFs with 5-Azacytidine, TSA, I-BET151 or GSK-J4 (Figs. S5b, 4d and Table S2), drugs which activated paternal *Cdkn1c* in mESCs (Fig. 2c). These treatments resulted in relatively minor changes in maternal *Cdkn1c-Fluc-lacZ* reporter expression in MEFs, as indicated by bioluminescence imaging of M5.3p cells (Fig. 4d, left). In contrast, BLI of P2.2p and P2.2i MEFs (Fig. 4d, right) revealed that paternal *Cdkn1c-Fluc-lacZ* reporter expression was significantly increased by TSA or GSK-J4 (as in mESCs), but not by 5-Azacytidine or I-BET151 (unlike in mESCs), with very similar responses seen in primary and immortalised cells. All treatments reduced the number of viable cells to some extent, with a particularly large reduction following GSK-J4 treatment (Fig. S5c). Detailed molecular analysis of *Kcnq1ot1* and allelic *Cdkn1c* mRNA expression in P2.2i MEFs confirmed that both TSA and GSK-J4 induced a significant increase in *Cdkn1c*, especially from the paternal allele, without affecting *Kcnq1ot1* levels (Fig. 4e). To discern whether paternal activation was transient or heritable, we imaged P2.2i MEFs 24 h after treatment, and again 24 h after drug removal (48 h). As shown in Fig. 4f, the impact of TSA declined following drug withdrawal, whereas the impact of GSK-J4 treatment on paternal *Cdkn1c* expression was retained at 48 h. Although the reversal with TSA was less pronounced, a similar result was observed for primary P2.2p MEFs (Fig. S5d). We previously noted that GSK-J4 treatment was associated with reduced cell numbers and apparent toxicity, and we found that treatment of MEFs generated a substantial increase in DNA damage, as measured by γH2AX staining (Fig. 4g and h, left). GSK-J4 treatment also induced high levels of DNA damage in mESCs (Fig. 4h, right and Fig. S5e), indicating that the sustained de-repression of paternal *Cdkn1c* in both cell types may be related to cellular stress and a likely block of cell-cycle progression.

## Discussion

In this study we have demonstrated that bioluminescent reporter genes can provide accurate, convenient and sequential temporal readouts of endogenous allelic expression and epigenetic regulation and disruption. We have performed a relatively small screen of selected inhibitors in the context of imprinting disruption; however, this approach should be scalable to larger scale screens, including automated imaging, to test and investigate epidrugs in a variety of contexts. Here, we used these tools to identify and investigate agents that relieve repression of paternally imprinted *Cdkn1c*. Several drugs, including 5-Azacytidine, HDAC inhibitors, BET inhibitors and an inhibitor of KDM6A/B, were found to relieve repression at the paternal *Cdkn1c* allele in mESCs, either selectively or by inducing biallelic effects. It was also interesting to note that inhibition of other factors previously associated with imprinting silencing, such as G9a and PRC2^23,51,52,78^, did not provoke reactivation, consistent with reports suggesting that these complexes play a greater role in extraembryonic tissues^49,52,79–81^.

Amongst the positive candidates identified, two general HDAC inhibitors (TSA and VPA) and a class I HDAC inhibitor (Romidepsin) induced paternal activation, providing robust evidence that ongoing histone deacetylation is required to maintain transcriptional silencing, consistent with previous literature^54,63–65,82^. More unexpectedly, we found consistent evidence that BET inhibition can disrupt *Cdkn1c* imprinting in mESCs, with two BET inhibitors (I-BET151 and GSK726) provoking significant increases in paternal *Cdkn1c* expression, whilst a third (GSK0858) produced a smaller (non-significant) increase. BET proteins are normally associated with activation rather than silencing^83^, suggesting a possible indirect mechanism, although we ruled out an effect on *Kcnq1ot1*. However, BET proteins have been implicated in silencing in some contexts^83^ and it has previously been reported that BET inhibitors can mimic the effects of HDAC inhibitors and act synergistically^82,84,85^, although the mechanisms remain unclear. Differences in *Cdkn1c* responsiveness between mESCs and fibroblasts indicate that functional repression of paternal *Cdkn1c* varies with cell type and stage of differentiation. Although histone deacetylation appears to be required for silencing in both cell types, BET inhibition specifically induces loss of silencing in mESCs with no impact in fibroblasts. Therefore, the mechanisms of de-repression appear to be varied and dependent on the activity of multiple chromatin-modifying enzymes.

Differential DNA methylation is central to establishing and maintaining imprinting, with two regulatory DMRs at the *Cdkn1c* locus (*KvDMR* and *sDMR*)^22,24,38–43^. However, treatment with 5-Azacytidine was not expected to relieve imprinted silencing of *Cdkn1c* in mESCs since the paternal allele is fully unmethylated at both DMRs in these cells, suggesting an indirect effect. The *sDMR* normally gains methylation between E6.5 and E9.5, which has been suggested to reinforce and help maintain silencing^24,43^. Although this may be important in vivo, immortalised MEFs maintain silencing of paternal *Cdkn1c* despite loss of *sDMR* methylation. This suggests that *sDMR* methylation is not always essential for maintaining *Cdkn1c* imprinting in somatic cells, and indeed the *sDMR* remains unmethylated during in vitro differentiation of mESCs^86^ and throughout normal development in humans^87–89^. Although we have identified treatments which are able to relieve paternal silencing, these generally exerted transient effects on *Cdkn1c*, and the underlying memory of imprinting appeared to be retained, even in the context of irreversible loss of pluripotency. Similarly, TSA and 5-Azacytidine treatment in utero, which is accompanied by erosion of DNA methylation of the *sDMR*, was previously shown to result in transient re-expression of paternal *Cdkn1c*^55^. Collectively, these data reinforce the idea that differential methylation at the *KvDMR* is central to retaining long-term paternal and maternal *Cdkn1c* memory, but that interference with other epigenetic mediators can transiently influence allelic expression and repression.

Amongst the agents identified as disrupting normal *Cdkn1c* imprinting, GSK-J4 was unique in provoking paternal *Cdkn1c* de-repression which was retained in both mESCs and MEFs 24 h after drug removal. GSK-J4 is an inhibitor of KDM6A/B^75^, enzymes which remove H3K27me3 and would normally be considered activators. This may suggest that KDM6A/B regulate *Cdkn1c* indirectly, or that the effects of GSK-J4 may be due to a lack of specificity to the KDM6 sub-family^90^. However, another explanation for our observations is that GSK-J4 induces significant levels of DNA damage^91^, exhibits noticeable toxicity, and has previously been implicated in provoking apoptosis^92^. This raises the possibility that the effect may in part be due to cellular stress and impacts on cell cycle progression, especially if division is required to re-establish normal imprinted silencing following drug removal. Irrespective of this, here we have shown how bioluminescent reporter cell lines for imprinted genes enable differential comparisons between maternal and paternal alleles, and how these new tools can be used to rapidly and reliably screen agents that interfere with epigenetic silencing.

## Methods

### Reporter cell lines

*Cdkn1c-FLuc-lacZ* reporter mouse mESC clones (B07 and H05, both male) were previously generated by Taconic Biosciences^55^. Mouse ESCs were cultured on 0.1% gelatin-coated plates in KnockOut DMEM medium (Gibco) supplemented with 20% FCS, non-essential amino acids, L-glutamine, penicillin/streptomycin, β-mercaptoethanol and 1000 U ml^−1^ leukaemia inhibitory factor. ESCs were cultured at 37 °C with 5% CO_2_ and split every 2–3 days.

MEFs were derived from E13.5–14.5 embryos from the previously described *Cdkn1c-Fluc-lacZ* mouse reporter line generated by Taconic Biosciences^55^, which was maintained on a 129S2/SvHsd background. Mouse work (solely for derivation of MEFs) was performed in accordance with the United Kingdom Animals (Scientific Procedures) Act (1986), under a UK Home Office project license, with approval from the Imperial College AWERB committee. Mice were housed in pathogen-free conditions on a 12 h light–dark cycle with regulated temperature (21 ± 2 °C) and humidity (55 ± 10%). To generate embryos inheriting the reporter maternally or paternally, reciprocal crosses of WT and heterozygous *Cdkn1c-Fluc-lacZ* animals were performed by setting up males with not more than three females. Morning plug checking was performed, with females considered E0.5 upon plug discovery. Pregnant dams were culled at E13.5–14.5 and embryos dissected into PBS. The head, heart and liver were removed, and a tissue sample was collected for genotyping. The remaining tissue was minced with a scalpel before adding 2 ml trypsin and transferring to a 15 ml falcon for incubation at 37 °C for 20 min, with regular mixing by pipetting. After addition of 6 ml of MEF medium (DMEM, 10% FCS, l-glutamine, penicillin/streptomycin, β-mercaptoethanol) and vigorous mixing by pipetting, the disaggregated cells were passed through a 70 µm filter, transferred to a T75 flask and cultured at 37 °C with 5% CO_2_ and 3% O_2_. Meanwhile, genotyping was performed on the corresponding tissue sample by isolating genomic DNA in lysis buffer (0.05 M Tris–HCl pH 8, 0.025 M EDTA, 0.031% SDS, 0.02 M NaCl, 80 μg/ml Proteinase K (Sigma-Aldrich)) at 50 °C with agitation. DNA was diluted 1:2 in 10 mM Tris–HCl pH 8 and the presence of the *Cdkn1c-Fluc-lacZ* reporter allele was detected by PCR analysis using 1 µl of diluted DNA (primer sequences are provided in Table 1). Clone P2.2 was male and P2.4 was female, whilst clones M5.3 and M5.6 were not sex genotyped. Primary reporter MEFs were used at early passage numbers (< 10) or were immortalised by splitting every three days and seeding 1.5 × 10^4^ cells per cm^2^ until growth increased (around passage 20–25)^93^. Immortalised MEFs continued to be split every 3 days.

**Table 1:** Primer sequences.

Application/target	Forward	Reverse
Genotyping		
*Cdkn1c-Fluc-lacZ*^55^	CTCCATGCGATCACAGTGG	CTTTGGATCCAGTGGACTGG
Sex^95^	CTGAAGCTTTTGGCTTTGAG	CCACTGCCAAATTCTTTGG
Bisulphite sequencing		
*KvDMR*^41^	TAAGGTGAGTGGTTTAGGAT	AATCCCCCACACCTAAATTC
*sDMR*^55^	AGTATAATGTAGTATTTTTAGT	AAAACTATACCCAACTCCATA
RT-qPCR		
*β-Actin*	CATCCGTAAAGACCTCTATGCCAAC	ATGGAGCCACCGATCCACA
WT *Cdkn1c*^29^	AGAGAACTGCGCAGGAGAAC	TCTGGCCGTTAGCCTCTAAA
*Cdkn1c-Fluc-lacZ*^55^	AGAGAACTGCGCAGGAGAAC	GTTCCATCTTCCAGCGGATA
Total *Cdkn1c*	TCCTCCCGTGGCATTAAAGG	CTTAGCTGCACCCCTACCAG
*Kcnq1ot1*^47^	TTGGATTACTTCGGTGGGCT	ACACGGATGAAAACCACGCT
*Oct4*	AGAGGATCACCTTGGGGTACA	CGAAGCGACAGATGGTGGTC

### Bioluminescent imaging

BLI was performed in standard multi-well tissue culture plates, or in black-walled 96-wp plates (Thermo Scientific Nunc), with details provided in the figure legends. D-Luciferin (PerkinElmer) was dissolved in H_2_0 at 30 mg/ml and added to medium at 150 μg/ml prior to BLI. Plates were immediately imaged using an IVIS Spectrum or IVIS Lumina Series III (both PerkinElmer) using Living Image software (v4.5 (Spectrum) or v4.7.4 (Lumina Series III), Caliper Life Sciences/PerkinElmer). Images were taken at field of view C (Spectrum) or D (Lumina Series III), with F/Stop 1, binning 8 and 180 s exposure, with the stage temperature set to 37 °C. For quantification of bioluminescent signal (Living Image software), a grid of regions of interest (ROIs) was drawn over the plate image and used to measure the average radiance from each well. Additional ROIs were drawn outside the plate area and used to subtract background signal.

### RT-qPCR

RNA was extracted with the RNeasy Mini kit (Qiagen) before DNase treatment using the TURBO DNA-*free* kit (Invitrogen). Reverse transcription was performed using Superscript III Reverse Transcriptase (Invitrogen) and random primers. Quantitative real-time PCR was performed on a CFX96 Real-Time System (Bio-Rad, CFX Manager v3.1) with QuantiTect SYBR Green Master Mix (Qiagen) in a 10 µl reaction volume, using primers listed in Table 1. Gene expression was normalised using *β-Actin* transcript levels.

### Western blotting

10^6^ mESCs were washed in PBS, snap frozen and stored at − 80 °C. For processing, cells were resuspended in 50 µl PBS before adding 50 µl 2 × Laemmli buffer (120 mM Tris–HCl pH 6.8, 4% SDS, 20% glycerol; without β-mercaptoethanol or bromophenol blue) and heated at 95 °C for 5 min. Protein quantification was performed with the Qubit Protein Assay Kit (Invitrogen) using 1 µl of sample. β-mercaptoethanol and bromophenol blue were added to samples to a final concentration of 10% and 0.001% respectively, and samples were diluted to 1 µl/µg with 1 × Laemmli buffer (60 mM Tris–HCl pH 6.8, 2% SDS, 10% glycerol, 10% β-mercaptoethanol, 0.001% bromophenol blue). Western blots were performed according to standard procedures by resolving 10 µg protein on a 10% acrylamide gel, semi-dry transfer onto a PVDF membrane, blocking with 5% milk in TBS-T, and incubating with primary then secondary antibodies for 1 h each at room temperature (anti-Lamin B1 (primary = Santa Cruz sc-6216, 1:5000; secondary = Invitrogen donkey anti-Goat IgG (H + L) Alexa Fluor 680 A-21084, 1:10,000) and anti-CDKN1C (primary = Abcam ab75974, 1:500; secondary = Invitrogen goat anti-Rabbit IgG (H + L) Alexa Fluor 680 A-21109, 1:10,000)). Fluorescent detection was performed using the LI-COR Odyssey CLx imaging system and Image Studio software (v4.0.21).

### Bisulphite sequencing

Genomic DNA was extracted from cells with the DNeasy Blood & Tissue Kit (Qiagen). Bisulphite modification of DNA was performed with the EZ DNA Methylation-Gold Kit (Zymo Research) according to the manufacturer’s recommendations. Regions within the *KvDMR* or the *Cdkn1c sDMR* were PCR amplified with TaKaRa EpiTaq HS (Takara), using primers that specifically recognize bisulphite-converted DNA (sequences are provided in Table 1). PCR products were separated by agarose gel electrophoresis and bands corresponding to the predicted size were excised and purified with a QIAquick Gel Extraction Kit (Qiagen). Purified products were ligated into the pJET1.2/blunt cloning vector using the CloneJET PCR Cloning Kit (Thermo Scientific), as per the manufacturer’s protocol, before heat-shock transformation into DH5α competent cells. Bacteria were plated onto LB/ampicillin plates and grown overnight at 37 °C. Colonies were picked (18–24 per sample) and expanded in 1 ml LB/ampicillin broth overnight at 37 °C. The following morning plasmids were purified with the Wizard SV 96 Plasmid DNA Purification System (Promega), according to the manufacturer’s protocol, and sent for Sanger sequencing with the PJET1-2R primer (GENEWIZ). Sequences with < 90% conversion of non-CpG Cs were excluded.

### Drug treatments

Drug inhibitors were gifted by GlaxoSmithKline or commercially obtained, with details provided in Supplementary Data S1. Cells were seeded at a density of 1 × 10^4^/cm^2^ for 4 h (mESCs) or 24 h (MEFs) before addition of vehicle control (water for VPA, DMSO for all other inhibitors) or inhibitors at the specified concentrations. Drug dilutions were made prior to addition, ensuring a consistent concentration of vehicle (0.1%) in all conditions. BLI was initially performed at 24 h. For further imaging of the same cells at 48 h, the medium was replaced and the cells were cultured for another 24 h, either with fresh drug (48 h treatment) or without drug (24 h treatment + 24 h removal).

### Cell viability assay

Cells were seeded at 4 × 10^4^/well in 48-wp format and treated with the indicated drugs for 24 h. CellTiter-Blue (Promega) was added to the medium for the final 4 h of treatment, according to the manufacturer’s instructions (80 µl added to 400 µl of medium). Fluorescent images were obtained using an IVIS Lumina Series III (PerkinElmer) and Living Image software (v4.7.4, PerkinElmer) with the following settings: lamp level high, excitation filter 560 nm, emission filter 620 nm, field of view D, F/Stop 1, binning 1, 1 s exposure. For quantification (Living Image software), a grid of ROIs was used to measure the average radiant efficiency from each well. A well containing only medium with CellTiter-Blue reagent was used to subtract background signal.

### γH2AX staining

Cells were cultured for 24 h on uncoated (MEFs) or gelatin-coated (mESCs) coverslips before addition of DMSO or 10 µM GSK-J4 and culturing for a further 24 h. Cells were fixed with 2% paraformaldehyde for 20 min, permeabilized with 0.4% Triton X-100 for 5 min and blocked for 30 min with 2.5% bovine serum albumin and 10% normal goat serum. Cells were stained with anti-Phospho-Histone H2A.X (S139) primary antibody (Cell Signalling Technology 9718 T, 1:100) at 4 °C overnight. Primary antibody was detected by incubating with donkey anti-rabbit IgG(H + L) Alexa Fluor 488 secondary antibody (Invitrogen A-21206, 1:400) for 1 h at room temperature. Samples were mounted in Vectorshield containing DAPI and imaged with a Leica SP5 II confocal microscope using the LAS-AF software (v2.7.3.9723). Representative images for visualisation were processed in Fiji/ImageJ (v1.53f51, http://imagej.nih.gov/ij), keeping the brightness and contrast for the γH2AX channel consistent between images of the same cell type. Image quantification was performed using a custom CellProfiler pipeline (v4.2.4, https://cellprofiler.org)^94^. Briefly, nuclei were segmented using the DAPI channel, followed by measurement of mean intensity per nucleus in the γH2AX channel. As an additional semi-quantitative comparison for mESCs, four characteristic patterns of staining were defined (illustrated in Fig. S5e; I = very low signal/few foci, II = some signal/foci, III = high signal/multiple foci, IV = very high uniform signal) and cells were manually counted and categorised.

### Calculations, graphs and statistical analyses

Microsoft Excel and GraphPad Prism (v9.4.1) were used for calculations. GraphPad Prism (v9.4.1) was used for all statistical analyses and for preparing graphs, with details provided in the figure legends.

## Supplementary Information

Supplementary Information 1.

Supplementary Information 2.

## Data Availability

All data generated during this study are included in the article and its supplementary files or are available from the corresponding author on reasonable request.
