# Supplementary material for: Drug-induced loss of imprinting revealed using bioluminescent reporters of Cdkn1c
Source: Sci Rep. 2023 Apr 6;13:5626. doi: 10.1038/s41598-023-32747-6 (PMC10079848; doi:10.1038/s41598-023-32747-6)
Supplement: Supplementary file 2 — Supplementary Information 2. [file 41598_2023_32747_MOESM2_ESM.pdf]

Supplementary Information

Drug-induced loss of imprinting revealed using bioluminescent reporters of *Cdkn1c*

Dimond, A. *et al.*

Supplementary Figures S1-S5

Supplementary Tables S1-S2

Source Data for Figure 1e

Supplementary References

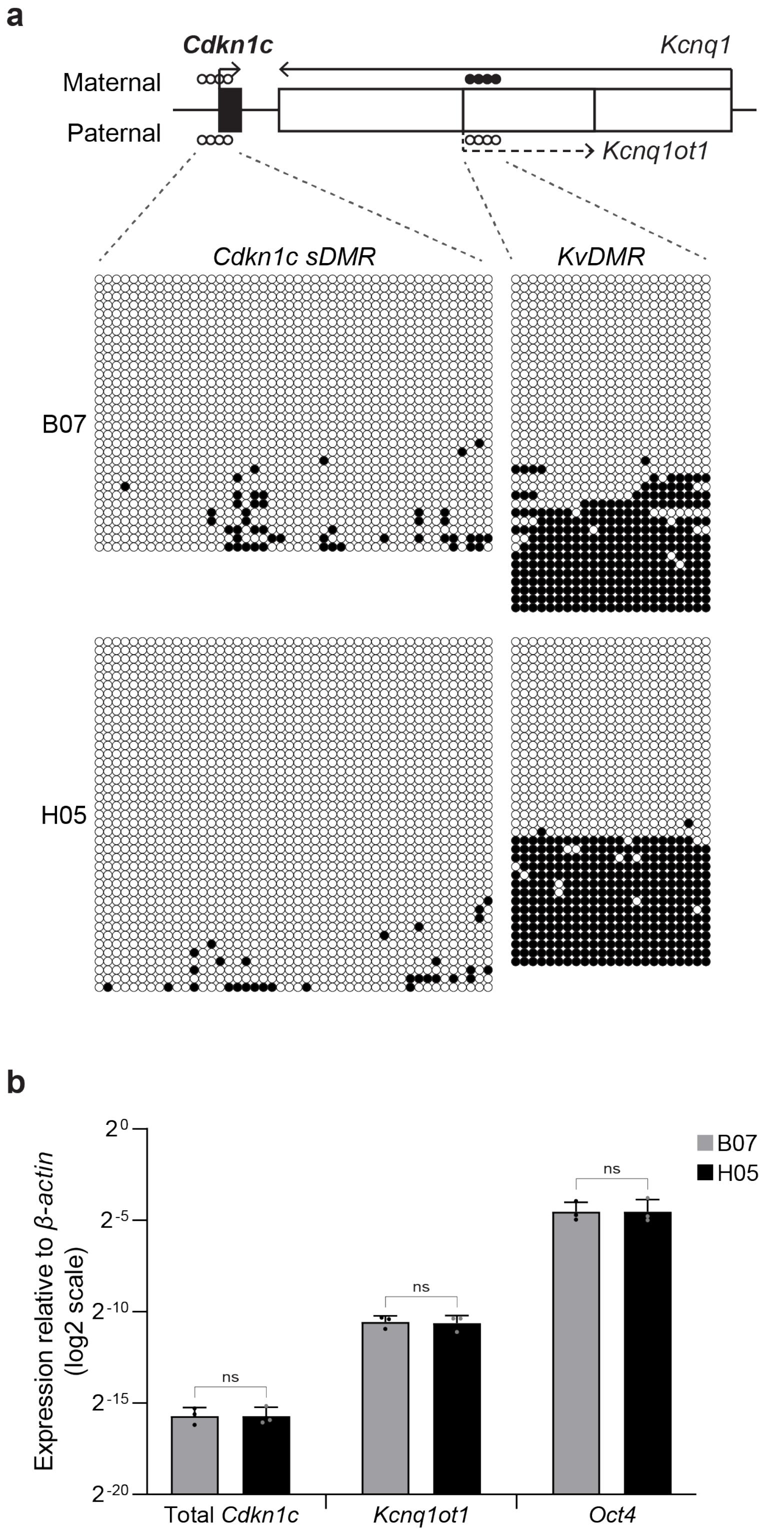

Supplementary Figure S1 | Characterisation of *Cdkn1c-Fluc-lacZ* reporter mESCs

a) Bisulfite methylation analysis at *Cdkn1c sDMR* and *KvDMR* in B07 and H05 mESCs. Each row represents an individual clone (closed circles=methylated CpGs, open circles=unmethylated CpGs). Data combined from two independent replicates. Both reporter lines show the expected patterns of methylation (illustrated above)[1,2]: mostly unmethylated at the *sDMR* and strands either unmethylated or fully methylated at the *KvDMR*.

b) Expression of total *Cdkn1c*, *Kcnq1ot1* and *Oct4* in B07 and H05 mESCs measured by RT-qPCR, relative to *β-actin* (n=3 independent replicates; bars indicate geometric mean; error bars represent geometric SD; unpaired two-tailed t-tests on delta-Ct values with Holm-Šídák's correction for multiple comparisons: ns=not significant).

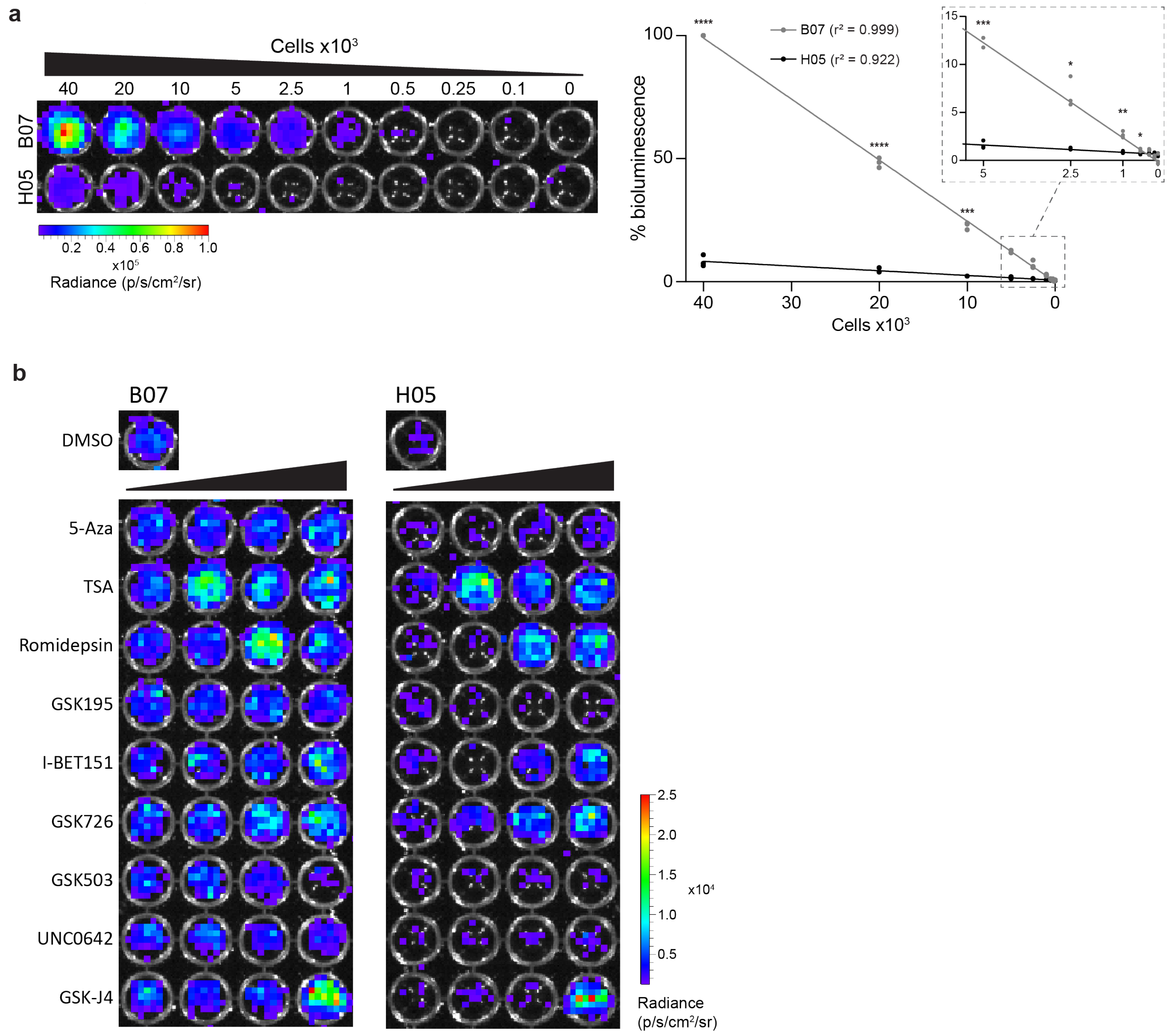

Supplementary Figure S2 | Potential of *in vitro* BLI reporter assays to be scaled up for high-throughput screening

a) BLI of defined numbers of B07 and H05 mESCs in black-walled 96-well plates, showing a representative image (left) and quantification (right). Quantification is relative to $40x10^3$ B07 mESCs for each dilution series (n=3 independent dilution series; simple linear regression; multiple two-sided t-tests with Holm-Šídák's correction comparing B07 to H05 for each dilution (****padj<0.0001, ***padj<0.001, **padj<0.01, *padj<0.05).

b) Proof of principle BLI of B07 and H05 mESCs in black-walled 96-well plates ($5x10^3$/well), treated for 24 h with increasing concentrations of the drugs indicated. All inhibitors were added at 10-fold increasing concentrations from 10 nM – 10 µM, except for Romidepsin which was added at 0.1 nM – 100 nM. Representative of two independent experiments. 5-Azacytidine, Class I HDAC inhibitors (TSA and Romidepsin), BET inhibitors (I-BET151 and GSK726) and GSK-J4 all visibly increased signal in H05 cells (paternal activation), similar to the data collected in larger plate formats.

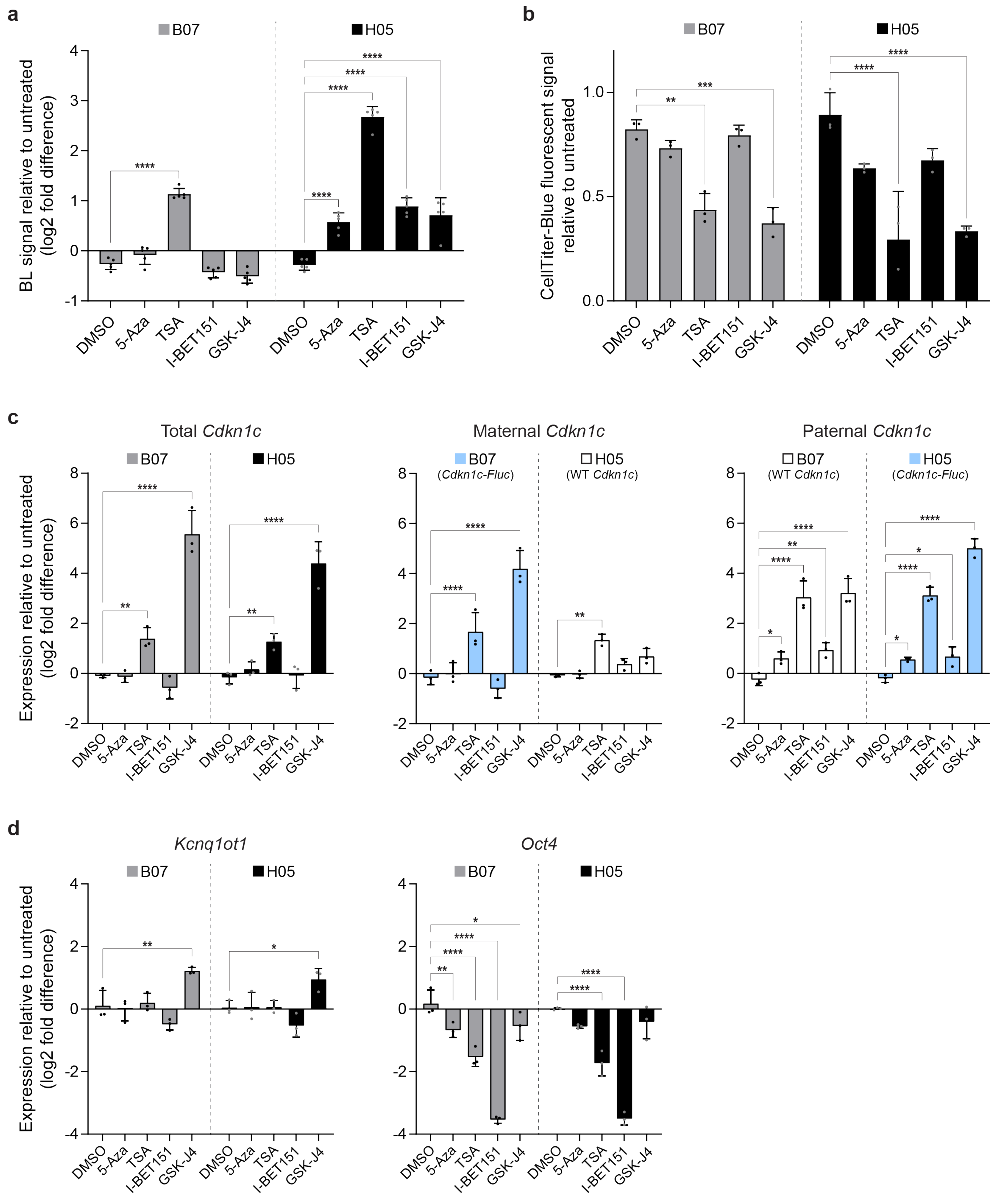

Supplementary Figure S3 | Validation of mESC reporter responses to selected drug treatments

a) Validation of B07 and H05 responses to 24 h treatment with selected inhibitors and concentrations (1 µM 5-Aza, 100 nM TSA, 10 µM I-BET151, 10 µM GSK-J4). Changes in bioluminescent signal are plotted as log2 fold differences relative to untreated cells (n=5 independent replicates; bars indicate mean; error bars represent SD; Two-Way ANOVA (Inhibitor $p<0.0001$, mESC clone $p<0.0001$, Interaction $p<0.0001$); results are shown for Holm-Šídák's multiple comparisons follow-up test comparing to DMSO: ****padj<0.0001).

b) CellTiter-Blue cell viability assay measurements for B07 and H05 mESCs following 24 h treatment with selected inhibitors and concentrations (1 µM 5-Aza, 100 nM TSA, 10 µM I-BET151, 10 µM GSK-J4), showing fluorescent radiant efficiency relative to untreated cells (n=3 independent replicates; bars indicate geometric mean; error bars represent geometric SD; Two-Way ANOVA on log2 transformed values (Inhibitor p<0.0001, mESC clone p=0.0709, Interaction p=0.4272); results of Holm-Šídák's multiple comparisons follow-up test comparing to DMSO are shown: ****padj<0.0001, ***padj=0.0006, **padj=0.0039).

c) RT-qPCR analysis of total (left), maternal (middle) and paternal (right) *Cdkn1c* expression in inhibitor-treated B07 and H05 mESCs (concentrations as in (a)). Expression is plotted as log2 fold difference relative to untreated cells, using *β-Actin* for normalisation (n=3 independent replicates; bars indicate mean; error bars represent SD; Two-Way ANOVAs (total *Cdkn1c* (Inhibitor p<0.0001, mESC clone p=0.5574, Interaction p=0.0841), maternal *Cdkn1c* (Inhibitor p<0.0001, mESC clone p=0.0013, Interaction p<0.0001), paternal *Cdkn1c* (Inhibitor p<0.0001, mESC clone p=0.0277, Interaction p=0.0007); results are shown for Holm-Šídák's multiple comparisons follow-up tests comparing to DMSO: ****padj<0.0001, **padj<0.01, *padj<0.05).

d) RT-qPCR analysis of *Kcnq1ot1* (left) and *Oct4* (right) expression in inhibitor-treated B07 and H05 mESCs (concentrations as in (a)). Expression is plotted as log2 fold difference relative to untreated cells, using *β-Actin* for normalisation (n=3 independent replicates; bars indicate mean; error bars represent SD; Two-Way ANOVAs (*Kcnq1ot1* (Inhibitor p<0.0001, mESC clone p=0.4804, Interaction p=0.9079), *Oct4* (Inhibitor p<0.0001, mESC clone p=0.8761, Interaction p=0.8421); results are shown for Holm-Šídák's multiple comparisons follow-up tests comparing to DMSO: ****padj<0.0001, **padj<0.01, *padj<0.05).

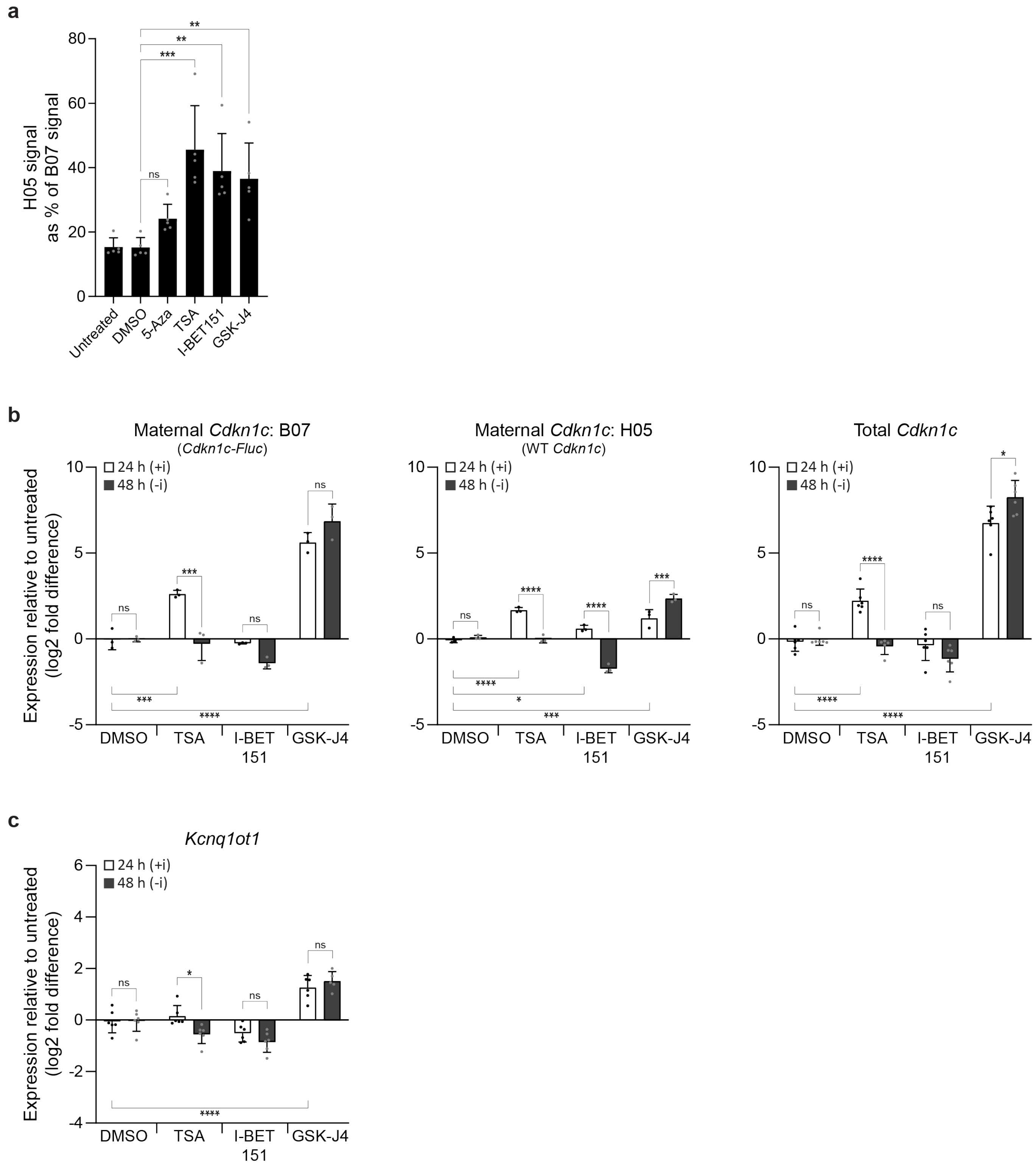

Supplementary Figure S4 | Reporter mESC responses to drug treatment and removal

a) H05 bioluminescent signal (representing paternal *Cdkn1c*) plotted as a percentage of B07 signal (representing maternal *Cdkn1c*) to identify which inhibitor treatments (1 µM 5-Aza, 100 nM TSA, 10 µM I-BET151, 10 µM GSK-J4) induce loss of imprinting by altering the relative contributions of each allele (percentages calculated from data shown in Figure S2a; n=5 independent replicates; bars indicate mean; error bars represent SD; One-Way ANOVA p<0.0001; results of Holm-Šídák's multiple comparisons follow-up test comparing to DMSO are shown: ***padj<0.001, **padj<0.01, ns=not significant).

b) RT-qPCR analysis of maternal *Cdkn1c* expression (left: *Cdkn1c-Fluc-lacZ* in B07 mESCs; middle: WT *Cdkn1c* in H05 mESCs; n=3 independent replicates) or total *Cdkn1c* expression (right; n=6 (3 B07 and 3 H05

independent replicates combined)) following inhibitor addition (24 h, +i), or inhibitor addition and removal (48 h, -i). Expression is plotted as log2 fold difference relative to untreated cells, normalised to *β-Actin* (bars indicate mean; error bars represent SD; Two-Way ANOVAs (B07 maternal (Inhibitor p<0.0001, Time p=0.0103, Interaction p=0.0001), H05 maternal (Inhibitor p<0.0001, Time p<0.0001, Interaction p<0.0001), total (Inhibitor p<0.0001, Time p=0.0431, Interaction p<0.0001); Holm-Šídák's multiple comparisons follow-up tests comparing all means: significant 24 h changes vs DMSO and results of 24 h vs 48 h comparisons are shown: ****padj<0.0001, ***padj<0.001, *padj<0.05, ns=not significant).

c) RT-qPCR analysis of *Kcnq1ot1* expression in reporter mESCs following inhibitor addition (24 h), or addition and removal (48 h). Expression is plotted as log2 fold difference relative to untreated cells, normalised to *β-Actin* (n=6 (3 B07 and 3 H05 independent replicates combined); bars indicate mean; error bars represent SD; Two-Way ANOVA (Inhibitor p<0.0001, Time p=0.1003, Interaction p=0.0345); Holm-Šídák's multiple comparisons follow-up test comparing all means: significant 24 h changes vs DMSO and results of 24 h vs 48 h comparisons are shown: ****padj<0.0001, *padj<0.05, ns=not significant).

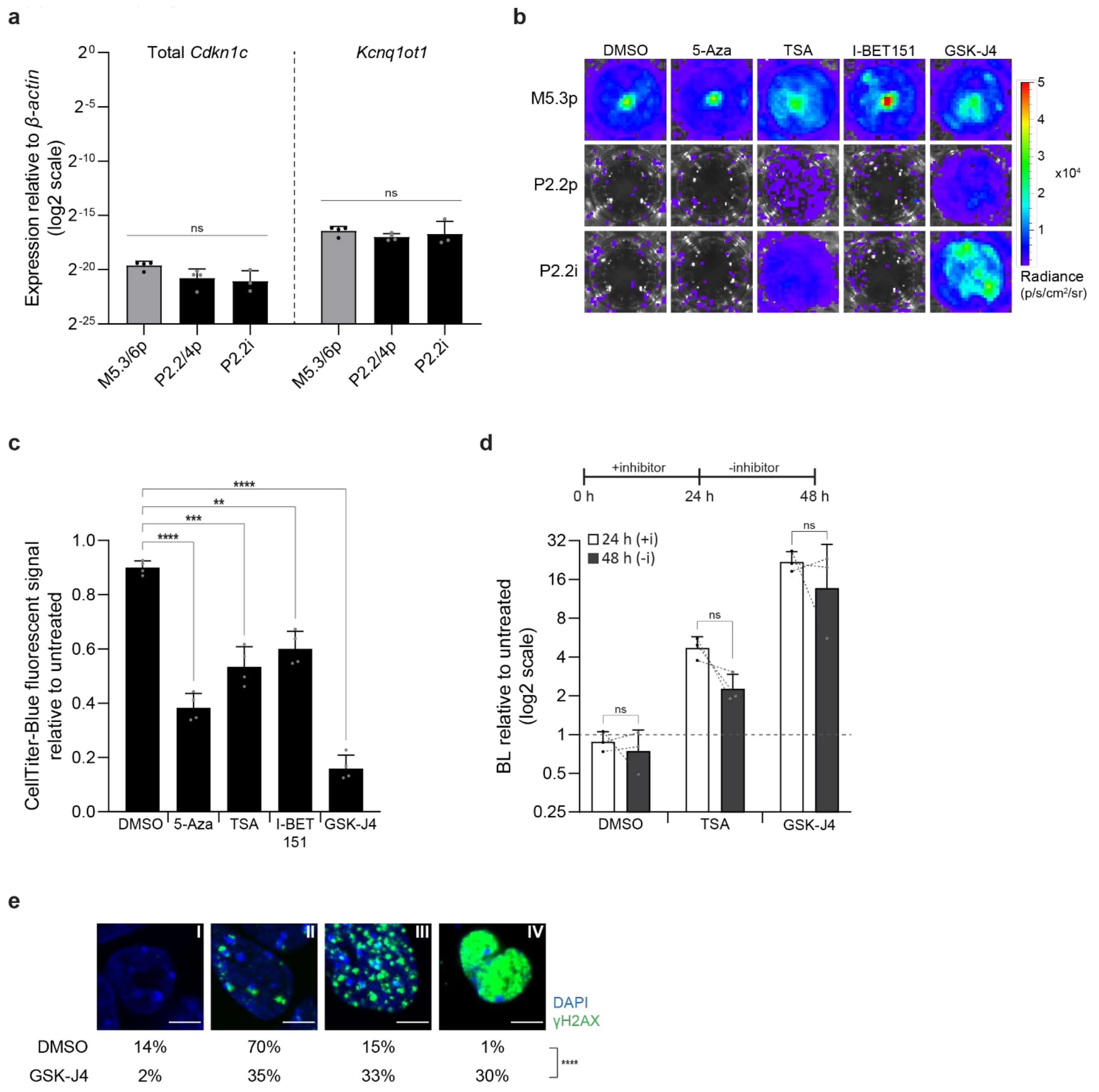

Supplementary Figure S5 | Gene expression and drug responses in reporter cells

a) RT-qPCR analysis of total *Cdkn1c* and *Kcnq1ot1* expression in primary and immortalised reporter MEFs, relative to *β-actin* (n=4 (primary) or 3 (immortalised); bars indicate geometric mean; error bars represent geometric SD; One-Way ANOVAs on delta-Ct values indicated no significant differences between reporter MEFs (total *Cdkn1c* p=0.0714, *Kcnq1ot1* p=0.5312)).

b) Bioluminescent images of primary and immortalised reporter MEFs treated for 24 h with the indicated inhibitors (all 10 µM) (48-wp format).

c) CellTiter-Blue cell viability assay measurements for P2.2i MEFs treated for 24 h with the indicated inhibitors (all 10 µM), showing fluorescent radiant efficiency relative to untreated cells (n=4 independent replicates; bars indicate geometric mean; error bars represent geometric SD; One-Way ANOVA on log2 transformed values (p<0.0001); results of Holm-Šídák's multiple comparisons follow-up test comparing to DMSO are shown: ****padj<0.0001, ***padj=0.0005, **padj=0.0023).

d) Paternal *Cdkn1c* activity in P2.2p MEFs was monitored by BLI following inhibitor treatment and removal (illustrated above). Bioluminescence signal was quantified relative to untreated cells at each timepoint

(n=3 independent replicates; bars indicate geometric mean; error bars represent geometric SD; dotted lines link repeated measurements at 24 h and 48 h within replicates; Repeated Measures Two-Way ANOVA on log2 transformed values (Inhibitor x Time $p=0.626$, Inhibitor $p<0.0001$, Time $p=0.0927$, Well replicate $p=0.8995$); results are shown for Holm-Šídák's multiple comparisons follow-up test comparing 24 h with 48 h: ns=not significant).

e) Images representing four categories of γH2AX staining (green) observed in H05 mESCs (I=very low signal/few foci, II=some signal/foci, III=high signal/multiple foci, IV=very high uniform signal). Scale bars represent 5 µm. The percentage of cells in each category after 24 h treatment with DMSO or 10 µM GSK-J4 is given below (n=374 (DMSO) and 359 (GSK-J4) cells; ****$p<0.0001$, Chi square test).

Supplementary Table S1 | Quantification of BL signal in reporter mESCs following inhibitor treatments

Target class	Drug	Specific target		24 h treatment					48 h treatment				
				Ctrl	→	→	→	→	Ctrl	→	→	→	→
DNA methylation	5-Azacytidine	DNMT1	B07	1.00	1.10	1.20	1.11	0.76	1.00	1.00	0.43	0.33	0.15
			H05	1.00	1.11	1.67	1.78	1.00	1.00	1.10	0.65	0.44	0.24
Histone deacetylases	TSA	Pan HDAC	B07	1.00	1.38	3.20	0.78	0.78	1.00	1.18	0.42	0.12	0.08
			H05	1.00	2.01	11.31	3.91	5.16	1.00	1.59	0.95	0.35	0.36
	VPA	Class I and IIa HDACs	B07	1.00	0.97	0.96	1.04	1.37	-	-	-	-	-
			H05	1.00	1.05	1.04	1.37	3.40	-	-	-	-	-
	Romidepsin	Class I HDAC	B07	1.00	0.97	1.44	2.14	0.85	-	-	-	-	-
			H05	1.00	1.03	2.11	11.07	4.50	-	-	-	-	-
	GSK195	Class IIa HDAC	B07	1.00	0.99	1.00	1.05	0.96	1.00	1.04	1.02	0.98	0.88
			H05	1.00	1.03	1.01	1.08	0.93	1.00	0.98	1.00	0.94	0.70
	GSK058	Class IIa HDAC negative control	B07	1.00	0.94	0.90	0.97	0.90	1.00	0.84	0.86	0.76	0.54
			H05	1.00	0.99	0.95	0.95	1.28	1.00	1.07	1.01	0.89	0.95
Histone acetyltransferases &	GSK077	CREBBP/EP300 (H3K27ac)	B07	1.00	1.04	0.97	1.06	0.86	-	-	-	-	-
acetyl binding			H05	1.00	0.91	0.99	0.94	0.82	-	-	-	-	-
	I-BET151	Pan BET (bromodomain)	B07	1.00	0.96	0.75	0.65	0.80	-	-	-	-	-
	(GSK151A)		H05	1.00	0.95	0.94	1.35	2.26	-	-	-	-	-
	GSK726	Pan BET (bromodomain)	B07	1.00	0.70	0.51	0.69	0.74	-	-	-	-	-
			H05	1.00	0.89	1.16	1.82	2.16	-	-	-	-	-
	GSK0858	Pan BET (bromodomain)	B07	1.00	0.67	0.62	0.66	0.53	-	-	-	-	-
			H05	1.00	0.91	1.00	1.28	1.22	-	-	-	-	-
	GSK0853	BRPF1 (bromodomain)	B07	1.00	1.03	1.07	1.21	1.23	-	-	-	-	-
			H05	1.00	0.92	0.98	1.11	0.93	-	-	-	-	-
	GSK311	BRPF1 less active control	B07	1.00	0.93	0.89	0.89	0.80	-	-	-	-	-
			H05	1.00	1.05	1.04	0.99	0.98	-	-	-	-	-
	GSK959	BRPF1 less active control	B07	1.00	1.04	1.15	1.18	1.07	-	-	-	-	-
			H05	1.00	0.99	1.06	1.10	0.94	-	-	-	-	-
	GSK602	BRD9 (bromodomain)	B07	1.00	0.98	0.97	1.12	1.02	-	-	-	-	-
			H05	1.00	1.00	1.00	1.05	1.14	-	-	-	-	-
	GSK814	ATAD2 (bromodomain)	B07	1.00	1.02	1.04	0.98	1.26	-	-	-	-	-
			H05	1.00	0.95	0.97	0.95	1.36	-	-	-	-	-
	GSK815	ATAD2 negative control	B07	1.00	1.00	0.96	1.00	1.28	-	-	-	-	-
			H05	1.00	0.97	0.98	1.06	1.11	-	-	-	-	-
	GSK2801	BAZ2A/B (bromodomain, rDNA	B07	1.00	0.98	1.01	1.00	0.98	-	-	-	-	-
		silencing)	H05	1.00	0.96	1.00	1.02	0.93	-	-	-	-	-
Histone methyltransferases	GSK343		B07	1.00	1.07	1.13	1.29	0.68	1.00	1.06	0.97	0.78	0.10
		PRC2 - EZH1/2 (H3K27me3)	H05	1.00	1.01	1.00	1.09	0.88	1.00	0.99	0.91	0.64	0.32
	GSK503		B07	1.00	1.06	1.04	1.22	1.33	1.00	0.96	0.94	0.90	0.17
		PRC2 - EZH1/2 (H3K27me3)	H05	1.00	0.99	1.02	1.10	1.03	1.00	0.95	0.92	0.77	0.29
	UNC1999		B07	1.00	1.00	1.04	0.95	0.24	1.00	0.99	0.92	0.59	0.10
		PRC2 - EZH1/2 (H3K27me3)	H05	1.00	1.05	1.04	1.02	0.65	1.00	1.02	0.91	0.62	0.40
	UNC0642	G9a/GLP (H3K9me2)	B07	1.00	0.87	0.88	0.84	0.42	1.00	0.95	0.80	0.73	0.09
			H05	1.00	0.90	0.92	0.91	0.57	1.00	0.90	0.87	0.83	0.24
	UNC0379	SET8 (H3K20me1)	B07	1.00	1.04	1.05	1.10	1.27	1.00	0.93	0.98	0.88	0.32
			H05	1.00	0.98	0.93	0.99	1.47	1.00	0.96	0.97	0.88	0.68
	SGC0946	DOT1L (H3K79me1,2,3)	B07	1.00	1.00	1.03	1.12	1.23	1.00	0.95	1.02	0.93	0.19
			H05	1.00	0.98	1.01	1.07	0.91	1.00	0.91	1.05	0.89	0.32
	GSK712A	PRMT1 (Arginine	B07	1.00	1.05	1.05	1.06	1.11	1.00	1.05	1.01	1.00	1.14
		methyltransferase)	H05	1.00	0.98	1.10	1.08	1.25	1.00	0.93	1.01	1.06	1.34
	GSK591	PRMT5 (Arginine	B07	1.00	1.03	0.99	0.95	0.96	1.00	0.97	0.92	0.75	0.63
		methyltransferase)	H05	1.00	0.82	0.69	0.58	1.17	1.00	0.95	0.83	0.68	0.79
Histone demethylases	GSK-J4	KDM6A/B (H3K27me3	B07	1.00	0.93	0.95	0.92	0.62	-	-	-	-	-
		demethylases)	H05	1.00	1.01	1.00	1.12	1.85	-	-	-	-	-
	GSK-J5	KDM6A/B negative control	B07	1.00	1.04	1.05	1.12	1.04	-	-	-	-	-
			H05	1.00	1.03	1.11	1.07	1.01	-	-	-	-	-
	GSK854	LSD1 (H3K4me2)	B07	1.00	1.13	1.20	1.16	1.09	-	-	-	-	-
			H05	1.00	1.08	1.02	1.06	1.23	-	-	-	-	-

Relative bioluminescent signal

Bioluminescent signal was quantified relative to vehicle control (Ctrl) in B07 and H05 *Cdkn1c-Fluc-lacZ* reporter mESCs, following 24 h or 48 h treatment with the indicated inhibitors (24- or 48-wp format). Inhibitors were tested at 10-fold increasing concentrations from 10 nM – 10 µM, except for VPA (1 µM – 1 mM) and Romidepsin (0.1 nM – 100 nM). All inhibitors were dissolved and diluted in DMSO, except VPA which was dissolved and diluted in water. Values (and shading intensity) indicate the geometric mean; n≥3 independent replicates; Two-Way ANOVA for each timepoint on log2 transformed values (24 h and 48 h results: Treatment (Inhibitor/mESC combination) $p<0.0001$, Concentration $p<0.0001$, Interaction $p<0.0001$); Holm-Šídák's multiple comparisons follow-up tests comparing to vehicle for each treatment: significant increases are highlighted in red (padj<0.05).

Supplementary Table S2 | Quantification of BL signal in reporter MEFs following inhibitor treatments

			24 h treatment				
**Drug**	**Specific target**		**Untr.**	**DMSO**	**100 nM**	**1 µM**	**10 µM**
5-Azacytidine	DNMT1	M5.3p	1.00	1.04	-	1.16	0.68
		P2.2p	1.00	0.97	-	1.17	0.68
		P2.2i	1.00	1.15	-	1.25	1.13
TSA	Pan HDAC	M5.3p	1.00	1.04	1.27	1.55	1.83
		P2.2p	1.00	0.97	1.19	3.88	5.49
		P2.2i	1.00	1.15	1.93	11.10	23.28
I-BET151 (GSK151A)	Pan BET (bromodomain)	M5.3p	1.00	1.04	-	-	1.23
		P2.2p	1.00	0.97	-	-	1.24
		P2.2i	1.00	1.15	-	-	1.16
GSK-J4	KDM6A/B (H3K27me3 demethylases)	M5.3p	1.00	1.04	-	-	1.00
		P2.2p	1.00	0.97	-	-	21.90
		P2.2i	1.00	1.15	-	-	20.22

Relative bioluminescent signal

Bioluminescent signal was quantified in *Cdkn1c-Fluc-lacZ* MEF reporter cells following 24 h treatment with the indicated inhibitors and concentrations, relative to untreated cells (48-wp format). Values (and shading intensity) indicate the geometric mean; n≥3 independent replicates; Two-Way ANOVA on log2 transformed values (Treatment $p<0.0001$, MEF line $p<0.0001$, Interaction $p<0.0001$); Holm-Šídák's multiple comparisons follow-up tests comparing to DMSO for each treatment: significant changes are highlighted in red (padj<0.05).

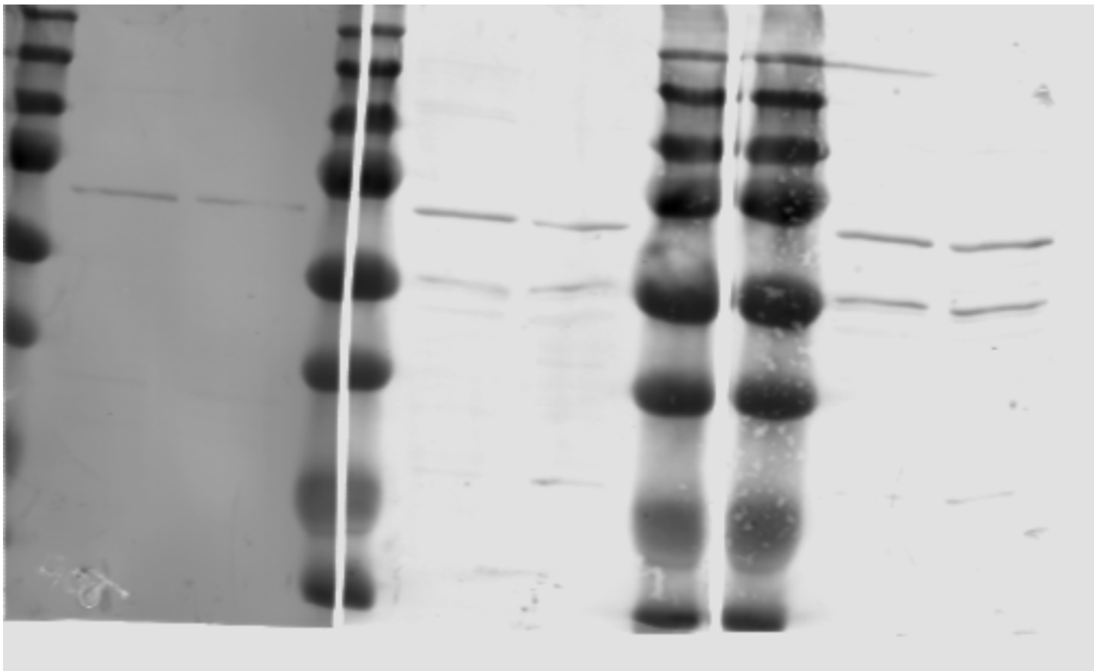

Source Data for Figure 1e | Uncropped and unprocessed blot corresponding to Figure 1e

Supplementary References

1. Wood, M. D. *et al.* Autonomous silencing of the imprinted Cdkn1c gene in stem cells. *Epigenetics* **5**, 214–21 (2010).
2. Bhogal, B., Arnaudo, A., Dymkowski, A., Best, A. & Davis, T. L. Methylation at mouse Cdkn1c is acquired during postimplantation development and functions to maintain imprinted expression. *Genomics* **84**, 961–70 (2004).
